# Fluorine substitution *vs.* central metal regulation: electronic properties and intrinsic reactivity of Au(iii)- and Ag(iii)-coordinated corroles from comprehensive DFT calculations

**DOI:** 10.1039/d6ra07081g

**Published:** 2026-10-12

**Authors:** Yilei Chen, Lin Dong

**Affiliations:** a YunCheng Campus, HeZe University HeZe 274015 China chenyilei@hezeu.edu.cn

## Abstract

Corrole macrocycles have broad application prospects in optoelectronics and biomedicine, yet the combined effects of central trivalent metal ions and fluorinated electron-withdrawing substituents on their electronic structures remain insufficiently understood. In this work, four Au(iii)/Ag(iii)-coordinated corrole complexes (Au-C, Au-C-F, Ag-C, Ag-C-F) were systematically studied *via* density functional theory (DFT) calculations at the M06-2X/def2-TZVP/SMD level. Multi-index bonding analysis methods, atomic charge population, conceptual DFT reactivity descriptors, frontier molecular orbital (FMO), LOL-π delocalization, multicenter bond order, NICS-AICD aromaticity evaluation and IRI weak interaction visualization were jointly adopted to reveal the dual modulation effect of metal centers and fluorine substitution. Bond order results prove N–Au coordination bonds possess stronger orbital overlap than N–Ag bonds; fluorine's strong electronegativity raises the positive charge of central metal ions and slightly weakens metal–nitrogen bonding interaction. CDFT and FMO analyses demonstrate fluorination widens the HOMO–LUMO gap, elevates ionization potential and electrophilicity index, and reduces nucleophilicity, which increases chemical hardness and kinetic resistance toward chemical perturbation while enhancing electron-accepting capacity. Aromaticity characterizations including multicenter bond order, NICS and AICD confirm Au(iii) corroles exhibit stronger macrocyclic aromaticity than Ag(iii) analogues, and fluorine substitution disperses delocalized π-electrons and weakens overall aromaticity. IRI mapping confirms fluorine perturbs intramolecular van der Waals interactions. This work systematically elucidates the independent and synergistic regulatory mechanisms of metal species and fluorine substituents, providing quantitative baseline theoretical references for corrole-based photofunctional materials and biosensing-related research.

## Abstract 2

Corrole macrocycles show promising applications in optoelectronics and biomedicine. The combined influences of central trivalent metal ions and fluorinated electron‑withdrawing substituents on their electronic structures remain poorly understood.

## Graphical abstract

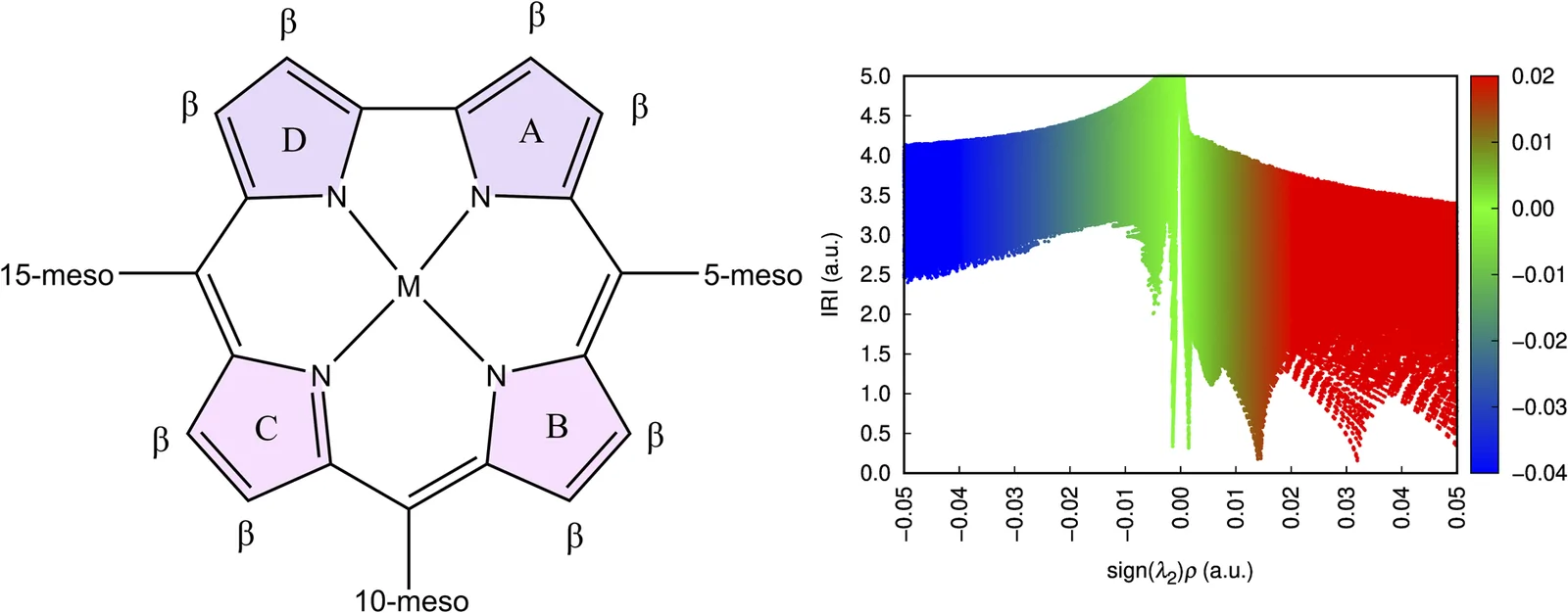

## 1 Introduction

As contracted tetrapyrrolic macrocyclic derivatives of porphyrins, corroles have garnered sustained research interest across multiple disciplines owing to their unique structural features and tunable physicochemical properties.^1–4^ Distinct from porphyrins, corroles possess one direct pyrrole–pyrrole linkage, only three *meso*-positions, a smaller central coordination cavity, and a trianionic ligand core upon full deprotonation, which endows them with a remarkable capability for stabilizing metal ions at high formal oxidation states.^5,6^ The fundamental chemistry of corroles, including synthetic advances, coordination behaviour and functional-material applications, has been comprehensively summarized in several authoritative review articles. Consequently, corroles have become a popular subject for both experimental measurements and quantum-chemical computational investigations.^7^

Compared with conventional porphyrins, the compressed macrocyclic skeleton of corroles creates a constrained central cavity, enabling the formation of characteristic metal-coordination complexes with diverse metal ions.^8^ Single-crystal X-ray diffraction experiments have offered detailed insights into their geometric parameters, π-electron delocalization characteristics, and substituent-dependent molecular planarity.^9^ Efficient one-pot synthetic protocols starting from pyrrole monomers have greatly improved synthetic accessibility and facilitated the preparation of various substituted corrole derivatives.^10^

Density functional theory (DFT) has emerged as a dominant theoretical tool for corrole-related studies. It permits atomic-level analysis of electronic structures, geometric conformations and reaction mechanisms, providing complementary theoretical support for experimental observations. Substituent modification can effectively redistribute macrocyclic electron density and tune frontier-molecular-orbital energies, thereby regulating redox potentials; DFT predictions generally show good consistency with electrochemical experimental data.^11,12^ Upon metal coordination, the corrole macrocycle often undergoes geometric distortion, whose magnitude is closely correlated with metal ionic radius, charge state and coordination mode. Computed structural parameters agree well with single-crystal diffraction results and help rationalize solubility and photophysical trends for metallocorrole complexes.^13–15^ Furthermore, DFT simulations are powerful for exploring catalytic reaction pathways, including locating intermediates and transition states and evaluating activation barriers, which helps uncover catalytic mechanisms and inspire rational catalyst design for corrole-based systems.^16^

Corrole-based materials exhibit promising prospects in photodynamic therapy, biological imaging, organic optoelectronic devices and electrochemical sensors, and substantial experimental progress has been achieved in these interdisciplinary directions.^17–20^ Among diverse metallocorrole families, trivalent gold(iii) and silver(iii) corroles are of particular fundamental interest because of their high formal oxidation states and open questions regarding metal–ligand covalency and ground-state electronic configurations.^21,22^

Over the past two decades, considerable experimental and theoretical efforts have been devoted to Au(iii)- and Ag(iii)-corroles, revealing their distinctive coordination chemistry and functional performance. Gold corroles obtained *via* Au(iii) metalation show high intrinsic stability and strong resistance toward demetallation.^23^ They have been explored as optical oxygen-sensing materials,^24,25^ chromophores for organic solar-cell devices,^26,27^ and efficient photosensitizers for singlet-oxygen generation.^24^ By contrast, silver corroles are chemically more labile and susceptible to demetallation under coordinating or reducing environments.^23,28^ Only very few examples of silver-corrole-mediated chemical reactivity have been documented so far; one notable recent case describes reactive-oxygen-species (ROS) detoxification activity.^29^ Given these obvious divergences between Au- and Ag-corroles, quantitative understanding of how the central metal and peripheral substituents jointly shape bonding, electronic structures and intrinsic reactivity is highly desirable for advancing corrole-based functional materials.

A critical electronic-structure complication specific to silver corroles is ligand non-innocence. Earlier literature has demonstrated that β-substituted silver corroles may not adopt a genuine closed-shell Ag(iii) ground state; instead, they can be described as antiferromagnetically coupled Ag(ii)-corrole radical-anion species originating from metal-to-ligand charge transfer.^30,31^ Computational evidence for such non-innocent electronic character has been reported for *meso*-substituted octa-fluorinated silver corrole, featuring obvious mixing between metal d(*x*^2^ −*y*^2^) orbital and corrole HOMO.^32^ Accordingly, spin-unrestricted broken-symmetry treatments are required in literature for reliable ground-state assignment of Ag-corrole systems. In the present study, we carried out wavefunction-stability analysis (Gaussian keyword ‘stable’) for our *meso*-unsubstituted silver-corrole models. The test confirms that the restricted closed-shell solution is stable and shows no tendency toward spontaneous symmetry breaking, which differs from literature *meso*-triaryl β-fluorinated Ag-corrole exhibiting ligand-non-innocent character. The corresponding computational details and full test outputs are provided in the main text and SI.

Despite the above advances, several knowledge gaps still remain. Existing theoretical investigations mostly focus on single-metal systems or unsubstituted/*meso*-triaryl corrole skeletons. Systematic side-by-side comparisons addressing the combined effect of trivalent Au^3+^/Ag^3+^ centers and strong electron-withdrawing β-fluorination are still scarce. It remains unclear to what extent metal identity and fluorine substitution cooperatively modulate metal–nitrogen bonding, atomic charge distribution, macrocyclic aromaticity, and intrinsic chemical reactivity. Such uncertainties hinder the rational development of corrole-related functional systems. It should also be noted that the overwhelming majority of modern corrole chemistry focuses on *meso*-triaryl-substituted derivatives; *meso*-aryl substituents can profoundly alter orbital energies and electronic properties, and hence results from *meso*-unsubstituted model systems cannot be directly transferred to *meso*-triaryl analogues without careful consideration. Nevertheless, *meso*-unsubstituted models offer a clean benchmark platform, which can isolate the intrinsic influences of metal center and β-substitution without *meso*-aryl perturbation.

To fill these gaps, four *meso*-unsubstituted corrole-metal complexes, namely unfluorinated gold corrole (Au-C), fluorinated gold corrole (Au-C-F), unfluorinated silver corrole (Ag-C), and fluorinated silver corrole (Ag-C-F), were constructed for comprehensive DFT calculations. Each category of computational analysis was designed to answer specific scientific questions:

(1) Metal–nitrogen bond-order and atomic-charge population analyses quantify how metal species and β-fluorination perturb M–N bonding strength and interfacial charge redistribution, reflecting the variation of metal–ligand covalency;

(2) Conceptual DFT global-reactivity descriptors are employed to quantitatively evaluate how metal replacement and fluorination regulate electrophilic and nucleophilic character of the macrocycle;

(3) Frontier-orbital analysis built upon the well-established Gouterman four-orbital model (which is also valid for corrole macrocycles^33–35^), together with LOL-π, multicenter bond-order, NICS and anisotropy-of-induced-current-density (AICD) ring-current calculations, jointly unravel metal- and substituent-driven modulation of π-electron delocalization and macrocyclic aromaticity;

(4) Interaction-region-indicator (IRI) visualization is applied to characterize how fluorine substitution reshapes intramolecular non-covalent weak interactions within corrole frameworks.

In this manuscript, comparative analyses across Au-C, Au-C-F, Ag-C and Ag-C-F were carried out to illustrate the independent and synergistic modulation originating from central-metal identity and β-fluorinated electron-withdrawing substituents, as shown in Scheme 1. The quantitative theoretical data reported herein serve as baseline references for future studies on substituted corrole analogues and help interpret the known experimental behaviors of Au/Ag corroles in photophysical and ROS-related functional scenarios.

**Scheme 1:**
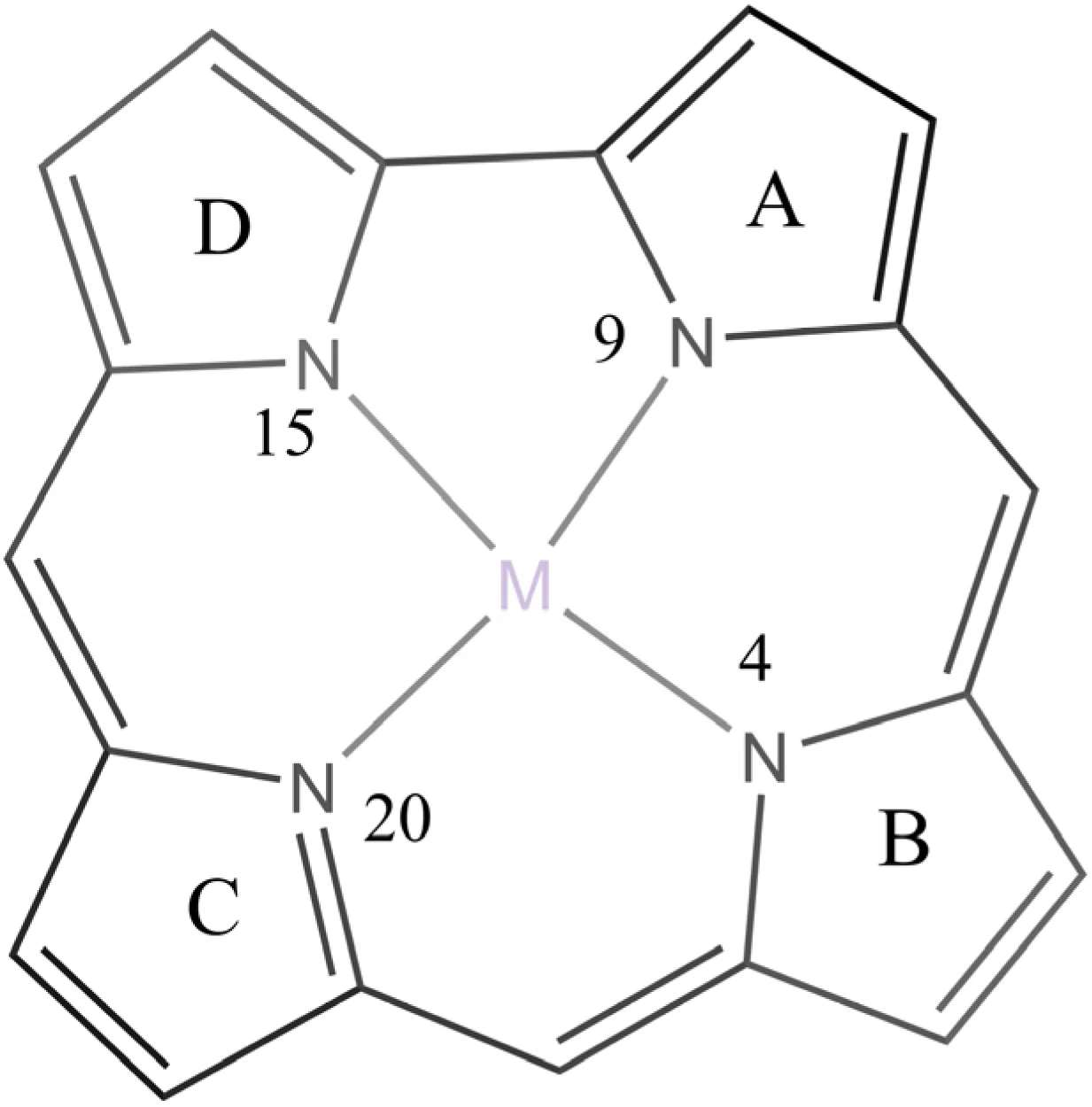
Structural diagram of the *meso*-unsubstituted metallocorrole skeleton, showing the four pyrrole rings (A–D) and coordinating nitrogen atoms coordinated to metal centre M.

## 2 Computational details

All geometric optimizations and frequency calculations of four corrole metal coordination complexes (Au-C, Au-C-F, Ag-C, Ag-C-F) were performed using the Gaussian program package.^36^ The M06-2X hybrid meta-generalized gradient approximation (*meta*-GGA) functional was selected to describe exchange–correlation interactions, combined with the third-generation empirical dispersion correction (D3, denoted as GD3) to accurately account for intramolecular van der Waals weak interactions.^37,38^ The all-electron triple-ζ valence polarized basis set def2-TZVP was adopted for all atoms in the system to ensure high accuracy of geometric and electronic property calculations.^39^

To simulate the actual experimental solvation environment, the SMD solvation model was introduced, with acetonitrile chosen as the implicit solvent. Geometry optimization was carried out without any symmetry constraints to obtain the stable ground-state conformation of each complex.^40^ Vibrational frequency calculations were performed on the optimised equilibrium structures to verify that all optimised geometries correspond to true potential-energy-surface minima; no imaginary vibrational frequencies were observed for any of the four target molecules.

Based on the fully optimized stable structures, a series of post-processing electronic property analyses were further conducted by Multiwfn,^41–43^ including Mayer/Wiberg/LBO bond order, multiple types of atomic charge population (ADCH, Hirshfeld, Mulliken, modified Mulliken), conceptual density functional theory (CDFT) global reactivity descriptors, frontier molecular orbital (FMO) energy and component decomposition, LOL-π π-electron localization analysis, multicenter aromatic bond order, NICS and AICD aromaticity evaluation, as well as IRI intramolecular weak interaction visualization.

For the four target complexes, standard restricted closed-shell DFT calculations were performed assuming formal Au(iii) and Ag(iii) d^8^ electronic configurations. Considering the reported ligand-non-innocent character for β-substituted *meso*-triaryl silver corroles in literature, wavefunction stability analyses (Gaussian keyword ‘stable’) were carried out for Ag-C and Ag-C-F to examine whether the restricted closed-shell SCF solution exhibits instability toward spin-symmetry breaking. Identical computational parameters (M06-2X-GD3/def2-TZVP, SMD-acetonitrile solvent model) were used for these stability tests. Wavefunction stability results are summarised in the SI. For Au-containing Au-C and Au-C-F complexes, only restricted closed-shell calculations were performed, consistent with the well-established closed-shell d^8^ ground-state of Au(iii) corroles.

## 3 Results and discussion

### 3.1 Bond-order

In this work, bond-order values for the four compounds were examined using three robust descriptors: Mayer, Wiberg, and Localized Bond Orbital (LBO) bond orders. The Mayer bond order evaluates shared electron pairs between atoms.^44^ The Wiberg bond order, an improved version of Mulliken-type analysis, properly accounts for heteroatom effects on adjacent bonds.^45,46^ The LBO descriptor is closely correlated with bond polarity, dissociation energy, and vibrational characteristics.^43^

The atom labels N4, N9, N15, N20 used in this table correspond to standard corrole numbering N22, N23, N21, N24 respectively. N21 and N24 belong to bipyrrole-type nitrogen atoms; N22 and N23 belong to dipyrrin-type nitrogen atoms. See Scheme 1 for four corrole nitrogen atom-numbering diagram.

Table 1 reveals consistent bond-order patterns between N atoms and Au or Ag atoms across the four compounds. In the Au-corrole system, N4–Au and N20–Au yield comparable bond-order values, while N9–Au and N15–Au show nearly identical magnitudes, reflecting equivalent bonding environments for these nitrogen–metal pairs. Similar trends are observed for the fluorinated Au-C-F as well as for the two silver-containing complexes.

**Table 1:** Bond orders between N, Au and Ag atoms in different compounds

Compound	Bonds	Mayer	Wiberg	LBO
Au-C	N4–Au	0.6012	0.7539	0.3825
	N9–Au	0.6253	0.7603	0.3962
	N15–Au	0.6253	0.7603	0.3962
	N20–Au	0.6012	0.7539	0.3825
Au-C-F	N4–Au	0.6014	0.7489	0.3840
	N9–Au	0.6189	0.7499	0.3968
	N15–Au	0.6189	0.7499	0.3968
	N20–Au	0.6014	0.7489	0.3840
Ag-C	N4–Ag	0.5697	0.7265	0.4928
	N9–Ag	0.5760	0.7285	0.5075
	N15–Ag	0.5760	0.7285	0.5075
	N20–Ag	0.5697	0.7265	0.4928
Ag-C-F	N4–Ag	0.5677	0.7220	0.4935
	N9–Ag	0.5678	0.7183	0.5091
	N15–Ag	0.5677	0.7183	0.5091
	N20–Ag	0.5677	0.7220	0.4935

Comparing metal–nitrogen bonding for different central metals, the N–Au bonds in Au-C and Au-C-F exhibit higher Mayer bond orders (0.6012–0.6253) than N–Ag bonds in Ag-C and Ag-C-F (0.5677–0.5760). Higher Wiberg bond-order values are also obtained for N–Au (0.7489–0.7603) relative to N–Ag bonds (0.7183–0.7285). Collectively, these results demonstrate stronger orbital overlap, greater electron-sharing character and higher intrinsic bonding robustness for N–Au coordination bonds compared with N–Ag bonds. Upon fluorination, slight decreases in metal–nitrogen bond-order magnitudes can be noticed for both Au and Ag systems, implying that fluorine-induced electron redistribution moderately weakens metal–nitrogen orbital interactions.

It should be noted that these bond-order descriptors are method-dependent quantities. Only relative trends under identical computational parameters are considered reliable, and small absolute numerical differences should not be over-interpreted.

### 3.2 Atomic charges

Before discussing atomic charge results, we note that wavefunction stability tests were performed for Ag-C and Ag-C-F to probe potential spin-symmetry-breaking behaviour associated with ligand-non-innocent electronic states. The restricted closed-shell wavefunctions are confirmed to be stable, with no indication of spontaneous spin-symmetry breaking for these *meso*-unsubstituted silver-corrole models. Therefore, all subsequent bonding and electronic-structure analyses are based on restricted closed-shell optimized geometries, and full wavefunction stability outputs are provided in the SI for reference.

Structural optimization reveals that all four compounds have *C*_2v_ symmetry, placing N4 and N20, as well as N9 and N15, in identical chemical environments with matching bond orders, confirmed by bond order analysis. Due to this symmetry, only N4 and N9 are analyzed for atomic charges. Four methods: atomic dipole moment corrected Hirshfeld population, Hirshfeld, Mulliken, and the Modified Mulliken method by Stout and Politzer were employed to calculate atomic charges,^47,48^ with results presented in Table 2.

**Table 2:** Atomic charges in four corrole metal coordination compounds

Compound	Atom	ADCH	Hirshfeld	Mulliken	Modified Mulliken
Au-C	Au	0.5375	0.5641	0.9165	0.7636
	N4	−0.1285	−0.1287	−0.3322	−0.6076
	N9	−0.1323	−0.1193	−0.3006	−0.6731
Au-C-F	Au	0.5762	0.6022	0.9606	0.9023
	N4	−0.1237	−0.1201	−0.3237	−0.5980
	N9	−0.1343	−0.1131	−0.2902	−0.6231
Ag-C	Ag	0.5381	0.5762	0.9135	0.7794
	N4	−0.1459	−0.1448	−0.3431	−0.5030
	N9	−0.1461	−0.1322	−0.3155	−0.5712
Ag-C-F	Ag	0.5769	0.6140	0.9645	0.8169
	N4	−0.1402	−0.1355	−0.3400	−0.5896
	N9	−0.1486	−0.1264	−0.3132	−0.5751

Atomic charge variations in metal atoms (ADCH method): in Ag-corrole, Ag's ADCH charge is 0.5381, increasing to 0.5769 in Ag-corrole-F. For Au-corrole and Au-corrole-F, Au have charges of 0.5375 and 0.5762, respectively. Fluorine increases metal atom charges due to its high electronegativity, which withdraws electron density, reducing electron density and increasing positive charge. Silver and gold compounds exhibit similar trends, with silver typically having slightly higher charges due to electronic structure differences.

Other methods (Hirshfeld, Mulliken, Modified Mulliken): these also show increased charges with fluorine addition. For example, Mulliken charges for Ag in Ag-corrole are 0.9135 and 0.9645 in Ag-corrole-F, while Au in Au-corrole and Au-corrole-F have charges of 0.9165 and 0.9606. Different methods produce varying charges due to their theoretical differences, with Mulliken based on molecular orbital theory and Hirshfeld partitioning electron density among atoms.

For N atoms (ADCH method): in Ag-corrole, N4 and N9 have charges of −0.1459 and −0.1461, while in Ag-corrole-F, they are −0.1402 and −0.1486. In Au-corrole and Au-corrole-F, the charges are −0.1459 and −0.1461, and −0.1237 and −0.1343, respectively. N atoms consistently exhibit negative charges across all compounds, with slight changes after fluorine addition. In silver compounds, the absolute values of N charges decrease, while in gold compounds, they vary due to fluorine's effect on electron cloud redistribution.

For N atoms (other methods: Hirshfeld, Mulliken, Modified Mulliken): charge trends are similar across methods, but numerical values differ significantly. The Mulliken method tends to yield larger absolute charge values due to its focus on atomic orbital contributions. Charge analysis indicates that atomic charge distribution is influenced by molecular structure and calculation method, affecting chemical properties like bond polarity and reactivity.

### 3.3 CDFT

The indices of conceptual density functional theory for four compounds are presented in Table 3.^49^

**Table 3:** Global reactivity indices of four compounds (eV)

	Au-C	Au-C-F	Ag-C	Ag-C-F
Ionization potential	6.5610	7.7285	6.7118	7.8693
Electron affinity	0.9677	2.0395	0.9732	2.4589
Electronegativity	3.7643	4.8840	3.8425	5.1641
Chemical potential	−3.7643	−4.8840	−3.8425	−5.1641
Hardness	5.5933	5.6889	5.7386	5.4104
Electrophilicity index	1.2667	2.0965	1.2864	2.4645
Nucleophilicity index	3.9283	2.7554	3.9713	2.7934
Cubic electrophilicity index	1.3485	2.1344	1.3921	2.4514
Electrophilic descriptor	0.8702	1.0461	0.9878	1.1267

Ionization energy and electron affinity: fluorination of the Ag-corrole and Au-corrole complexes significantly increases both the ionization energy and electron affinity. For instance, in Ag-corrole, fluorine increases ionization energy from 6.7118 eV to 7.8693 eV and electron affinity from 0.9732 eV to 2.4589 eV, enhancing electron binding and capture due to fluorine's high electronegativity.

Electronegativity and chemical potential: fluorine addition increases electronegativity and decreases chemical potential. In Au-corrole, electronegativity rises from 3.7643 eV to 4.8840 eV, and chemical potential decreases from −3.7643 eV to −4.8840 eV. This indicates that electrons tend to concentrate around more electronegative atoms, which contributes to increased chemical hardness and kinetic resistance toward chemical perturbation.

Hardness indicates a molecule's resistance to electron cloud deformation, while the electrophilicity index measures its electron-accepting tendency. Ag-corrole-F and Au-corrole-F exhibit little change in hardness but a significant rise in electrophilicity index compared to Ag-corrole and Au-corrole, with Ag-corrole's index increasing from 1.2864 eV to 2.4645 eV, suggesting fluorine enhances electrophilicity in reactions.

The nucleophilicity index decreases upon fluorination, indicating reduced electron donation, while the cubic electrophilicity index and electrophilic descriptor increase. For Au-corrole, the nucleophilicity index falls from 3.9283 eV to 2.7554 eV, the cubic electrophilicity index rises from 1.3485 eV to 2.1344 eV, and the electrophilic descriptor increases from 0.8702 eV to 1.0461 eV, confirming the enhanced electrophilic character.

In summary, the addition of fluorine atoms markedly alters the CDFT parameters of the compounds, affecting their electronic structures and chemical properties.

### 3.4 FMO

The frontier molecular orbitals illustrate the relationship between molecular reactivity and electronic structure, as shown in Fig. 1.

**Fig. 1:**
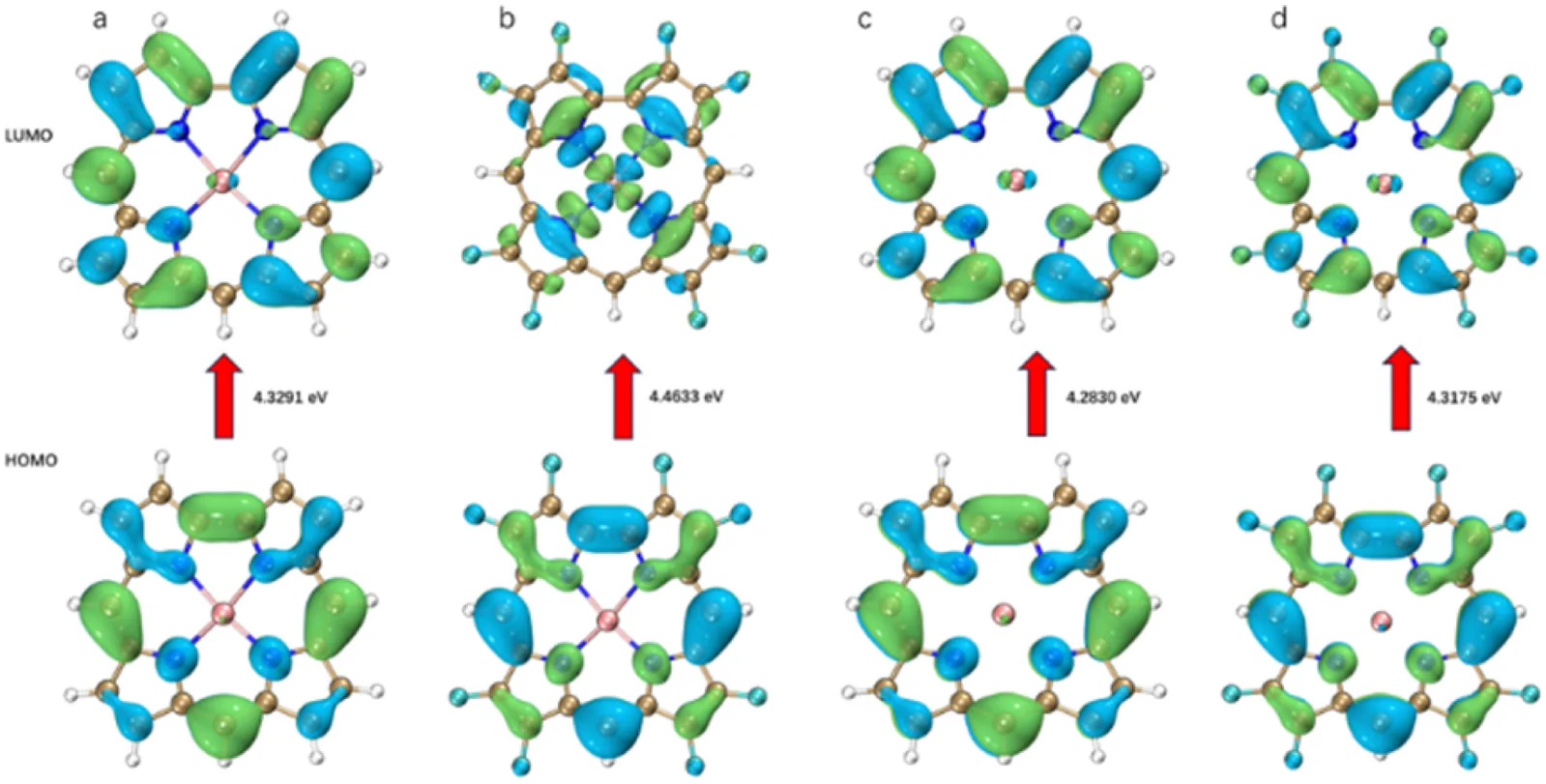
HOMO–LUMO gap of corrole metal coordination compounds. (a) Au-C; (b) Au-C-F; (c) Ag-C; (d) Ag-C-F.

A comparative analysis of Au-corrole and Au-corrole-F compounds reveals that the incorporation of fluorine atoms results in a decrease in both the Highest Occupied Molecular Orbital (HOMO) and Lowest Unoccupied Molecular Orbital (LUMO) energies. This finding indicates that fluorine substitution modifies the electronic configuration of the molecule and lowers the overall molecular-orbital energies. For the Ag-corrole/Ag-corrole-F pair, β-fluorination similarly decreases HOMO and LUMO orbital energies, which corresponds to higher chemical hardness and greater kinetic resistance towards chemical perturbation.

The energy gap (Δ*E*) correlates with molecular chemical hardness: a smaller Δ*E* generally corresponds to elevated chemical reactivity, whereas a larger Δ*E* points to enhanced kinetic resistance against chemical perturbation. The Au-corrole-F compound has a Δ*E* of 4.4633 eV, higher than that of Au-corrole (4.3291 eV), corresponding to elevated chemical hardness and enhanced kinetic resistance toward chemical perturbation. Similarly, the Ag-corrole-F compound's Δ*E* of 4.3175 eV is slightly higher than the Ag-corrole compound's 4.2830 eV, pointing to increased chemical hardness upon fluorination.

The Au-C compound has slightly lower HOMO and LUMO energies and a slightly higher Δ*E* than the Ag-corrole compound, which corresponds to greater chemical hardness and stronger kinetic resistance against chemical perturbation originating from the distinct electronic configurations of gold and silver.

The HOMO–LUMO gap primarily reflects the chemical hardness of a molecular system, rather than serving as a direct metric for relative thermodynamic stability. Larger HOMO–LUMO gap values correlate with enhanced kinetic resistance towards chemical perturbation and reactivity, but cannot be used alone to judge thermodynamic stability of these complexes.

### 3.5 π-Electrons population

LOL-π (Localized Orbital Locator for π electrons) analysis was performed for the four compounds.^50^ The results are shown in Fig. 2.

**Fig. 2:**
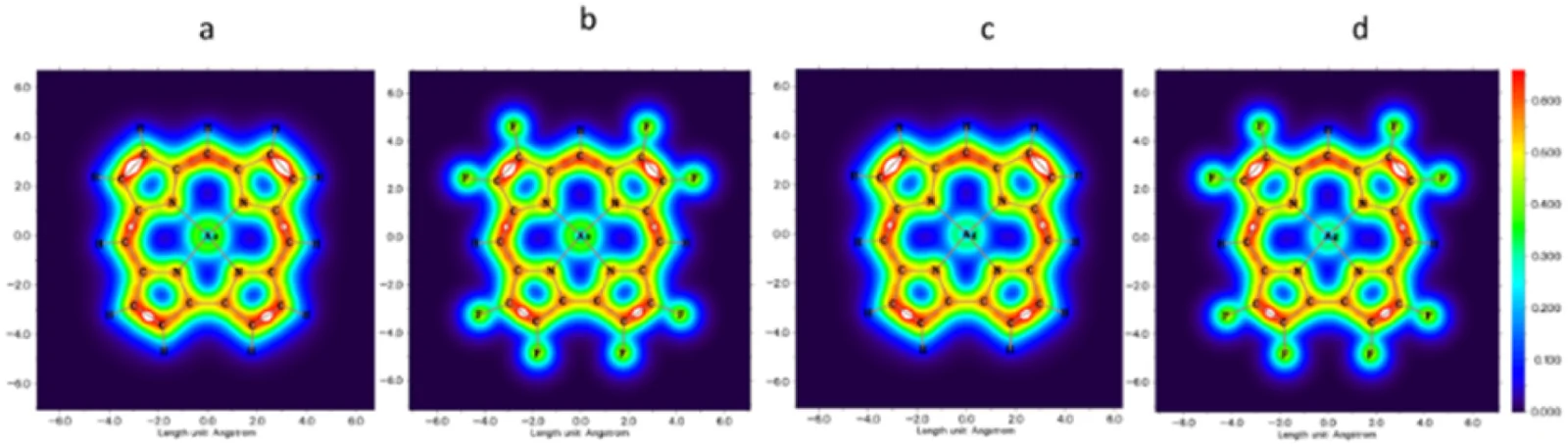
LOL-pi analysis for the four compounds, (a) Au-C; (b) Au-C-F; (c) Ag-C; (d) Ag-C-F.

This analysis examines π-electron delocalization, with a planar color-filled map at 1.2 bohr above the corrole plane providing clearer visualization than an isosurface map. Red paths show π-electron delocalization, influenced by the electronic structures of Au^3+^ ([Xe] 4f^14^5d^8^) and Ag^3+^ ([Kr] 4d^8^) and coordinated nitrogen atoms. In Au-corrole, Au^3+^'s 5d electrons interact with the corrole's π-electrons, leading to π-electron cloud accumulation near Au^3+^. Delocalization paths form a stable network along bonds and the corrole ring. Introducing fluorine in Au-corrole-F disrupts this balance, decreasing π-electron cloud density near fluorine and bending paths towards Au^3+^, while increasing density away from fluorine, creating distinct delocalization patterns.

In the Ag-corrole compound, Ag^3+^'s low nuclear charge allows π-electrons to delocalize freely across the corrole ring, with a uniform electron cloud indicating uniform electron distribution. In the Ag-corrole-F compound, fluorine reduces nearby π-electron density, and Ag^3+^'s weak electron-binding ability causes significant density changes far from fluorine, altering delocalization paths. Analyzing these paths, along with metal ions and nitrogen atoms, provides insights into π-electron delocalization patterns, aiding research on electronic structures, chemical bonds, and reaction mechanisms at a microscopic level.

Note that visual differences among these LOL-π plots are subtle upon naked-eye inspection. The π-electron redistribution induced by fluorination is mainly supported by the quantitative multicenter bond-order data, further complemented by subsequent AICD-NICS aromaticity results.

Fig. 2 provides only qualitative visual auxiliary information for π-electron distribution; quantitative assessment of π-electron delocalization is primarily based on multicenter bond-order data summarised in Table 4, and numerical LOL-π values can be read directly from the accompanying colour-bar scale of Fig. 2.

**Table 4:** Multicenter bond order analysis of rings in the four compounds

	Ring A	Ring B	Ring C	Ring D
Au-C	0.0286	0.0402	0.0401	0.0279
Au-C-F	0.0255	0.0340	0.0340	0.0245
Ag-C	0.0299	0.0423	0.0412	0.0293
Ag-C-F	0.0266	0.0359	0.0350	0.0257

A multicenter bond order analysis was conducted on the four rings (A, B, C, and D) of the compounds Au-corrole, Au-corrole-F, Ag-corrole, and Ag-corrole-F to investigate changes in aromaticity after metal coordination, as shown in Table 4.

The uncoordinated corrole structure is aromatic due to the delocalization of 18 π electrons. Metal coordination alters this electron distribution, impacting aromaticity. In the Au-corrole compound, rings B and C have high bond orders (0.0402 and 0.0401), indicating enhanced electron delocalization and aromatic stability due to interaction with Au^3+^'s 5d electrons. Rings A and D have lower bond orders, contributing less to aromaticity.

Upon formation of the Au-corrole-F compound, bond orders in all rings decrease; for instance, ring A's bond order drops from 0.0286 to 0.0255. Fluorine's strong electronegativity disrupts the electron cloud, reducing electron delocalization and weakening aromaticity.

In the Ag-corrole compound, rings B and C have higher bond orders of 0.0423 and 0.0412, respectively, due to Ag^3+^'s weaker electron-withdrawing ability, which enhances electron delocalization and maintains aromaticity. Rings A and D, with lower bond orders, contribute less.

When the Ag-corrole-F compound forms, bond orders in all rings decrease again, with ring A's bond order falling to 0.0266. Fluorine alters the electron cloud, reducing delocalization and weakening aromaticity.

Trivalent gold or silver coordination and fluorine atom introduction can alter the electron cloud distribution and multicenter bond orders, affecting aromaticity.

### 3.6 Molecular orbital component decomposition

A composition analysis of key molecular orbitals is summarized in Table 5, showing the percentage contributions of Au, Ag, and N atoms to HOMO, HOMO−1, HOMO−2, LUMO, LUMO+1, and LUMO+2 orbitals for all four complexes.

**Table 5:** The composition percentages of molecular orbitals

	Atoms	HOMO	HOMO−1	HOMO−2	LUMO	LUMO+1	LUMO+2
Au-C	Au	2.091	0.659	9.183	0.979	1.620	44.143
	N	13.207	7.189	29.553	7.741	9.406	36.840
Au-C-F	Au	1.838	0.687	7.729	1.015	1.777	42.437
	N	12.567	7.571	25.815	8.238	10.784	34.072
Ag-C	Ag	2.096	0.584	4.536	0.814	41.784	1.402
	N	13.658	7.014	30.586	7.615	40.361	9.348
Ag-C-F	Ag	1.849	0.600	3.697	41.476	0.844	1.509
	N	13.019	7.387	26.330	38.685	8.107	10.662

For the Au-containing corroles, Au makes a considerable contribution (44.143%) to the LUMO+2 orbital of Au-C, while nitrogen dominates the HOMO-2 orbital (29.553%). After fluorine substitution in Au-C-F, the orbital contributions of Au and N are moderately reduced for these frontier orbitals. Such orbital-component features highlight that high-energy unoccupied orbitals of Au-corrole are heavily involved with gold-centered orbital character.

Distinct orbital-contribution patterns are found for Ag-centered analogues. In Ag-C, Ag and N contribute substantially to the LUMO+1 orbital (41.784% and 40.361%, respectively). For fluorinated Ag-C-F, large Ag and N contributions shift toward the LUMO orbital instead. This sharp difference between Au- and Ag-corroles originates from the distinct d-electron configurations of Au(iii) and Ag(iii), which reshapes the metal contribution position within the frontier-orbital manifold.

Fluorine substitution modulates the orbital percentage of metal and pyrrolic-nitrogen atoms for occupied frontier orbitals in both series. It reduces the metal and nitrogen proportions in occupied frontier orbitals and rearranges the orbital composition of low-lying unoccupied orbitals. These orbital-component differences partially rationalize the divergent electronic properties and chemical reactivity between Au- and Ag-corrole complexes.

It is worth noting that HOMO−2 and LUMO+2 lie outside the classic Gouterman four-orbital manifold which dominates low-energy optical excitations of corrole systems. Therefore, detailed discussion for HOMO−2 and LUMO+2 is moved to SI, and our main-text analysis focuses on the four key frontier orbitals (HOMO−1, HOMO, LUMO, LUMO+1).

### 3.7 NICS

NICS is defined as the negative isotropic chemical shielding at the geometric center of a ring's heavy atoms, measured in ppm. It is termed “nucleus-independent” because the point of measurement is not at an atomic nucleus. For aromatic systems, NICS values are negative. This study calculates and presents NICS maps from two angles: 1 Å above the ring and from the side, as shown in Fig. 3.

**Fig. 3:**
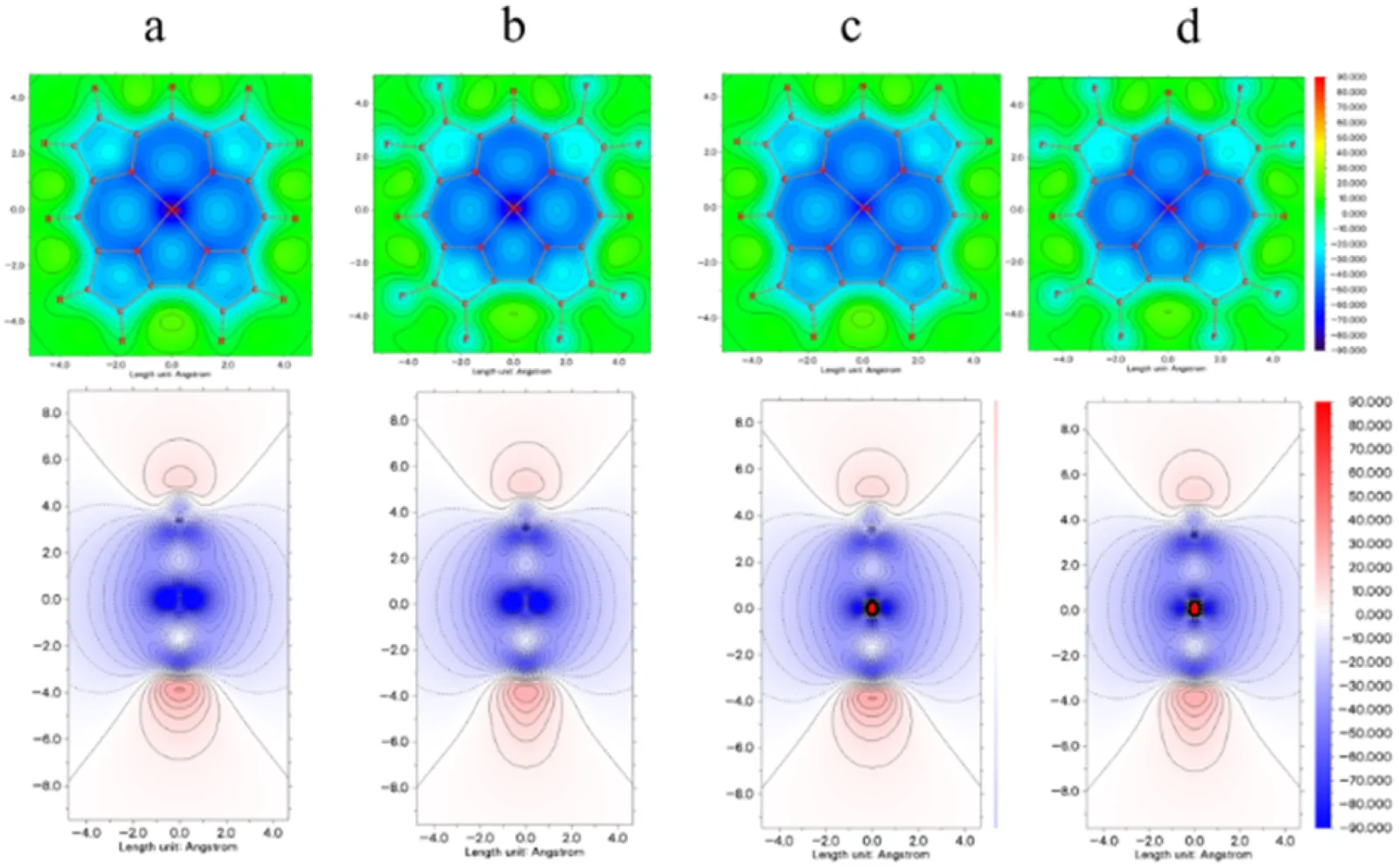
NICS diagrams of four compounds from different perspectives (1 Å above the macrocycle plane and side-view projection). Negative NICS values indicate aromatic features. (a) Au-C; (b) Au-C-F; (c) Ag-C; (d) Ag-C-F.

Nevertheless, for macrocyclic systems coordinated with transition-metal ions, local magnetic current densities originating from the metal center may interfere with NICS outputs and complicate aromaticity interpretation, as emphasized in recent literature.^51^ Therefore, we combined NICS calculations with AICD ring-current simulations to achieve a more reliable aromaticity evaluation.

It is well-documented that single-point NICS probes located directly above the metal centre can be strongly perturbed by local metal-centred magnetic currents, which may mask macrocyclic aromatic ring-current contributions.^51^ For this reason, aromaticity conclusions in this work are not based solely on single-point NICS values over the metal atom. Instead, we combine grid-sampled NICS maps distributed over the whole macrocycle, multicenter bond-order indices, and AICD ring-current streamlines to jointly evaluate corrole aromatic character.

The AICD plots for four corrole complexes are presented in Fig. 4, where the streamlines visualize the induced ring-current pathways over the corrole macrocycle. Obvious diatropic (clockwise) ring-current can be observed for all four complexes, confirming their aromatic nature. Qualitatively, the AICD streamline patterns suggest more prominent diatropic ring-current features for the Au-C complex. Upon β-fluorine substitution, the visual density of aromatic ring-current streamlines appears reduced for Au-C-F. We emphasise that Fig. 4 only provides qualitative visual information; no numerical quantitative values for ring-current magnitude can be extracted from these streamline plots. Our aromaticity conclusions are primarily supported by multicenter bond-order indices and grid-sampled NICS results, while AICD streamlines serve only as supplementary qualitative visual evidence.

**Fig. 4:**
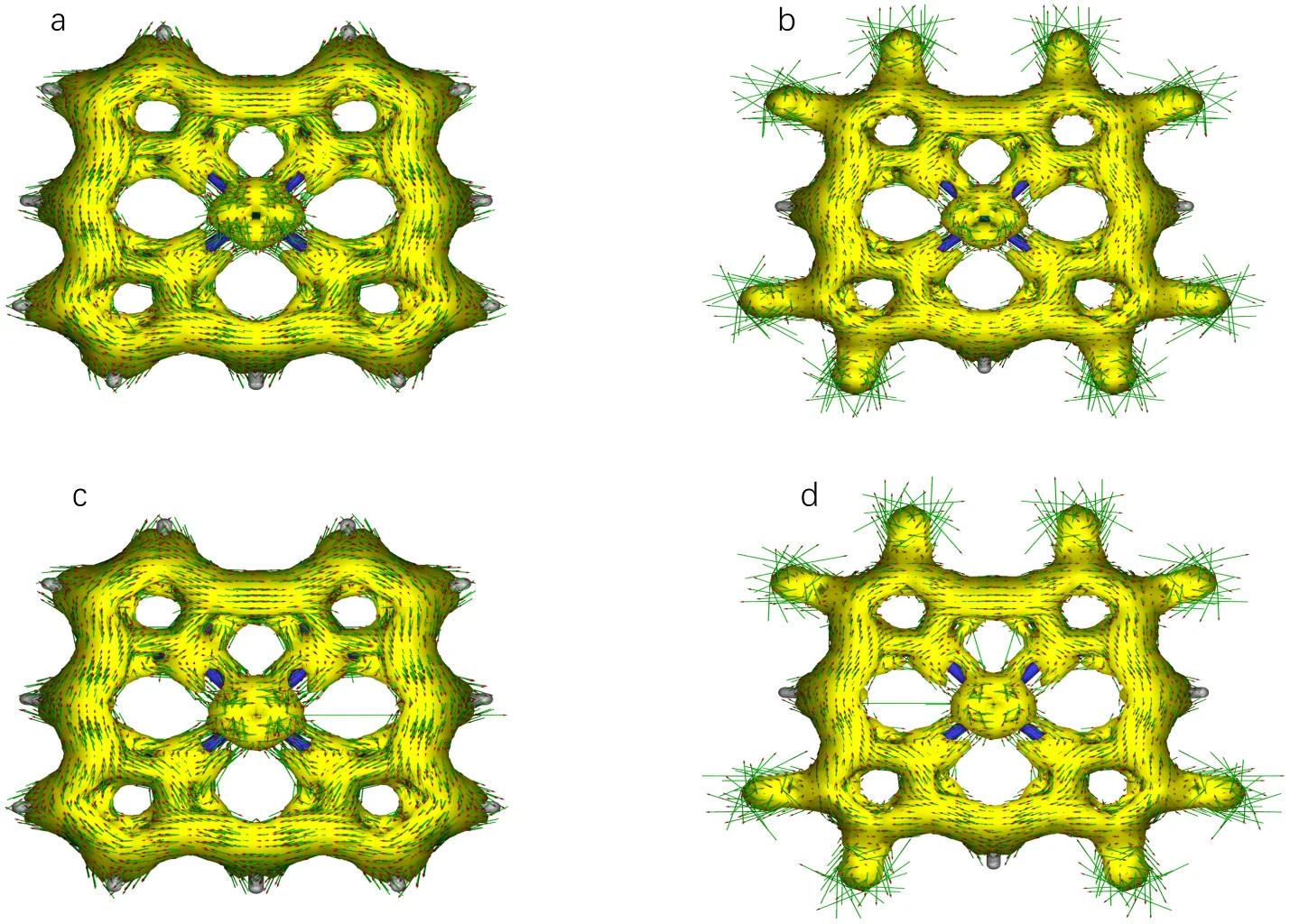
AICD plots showing induced-current-density streamlines of four corrole complexes. (a) Au-C; (b) Au-C-F; (c) Ag-C; (d) Ag-C-F. Clockwise diatropic streamlines represent aromatic ring-current character.

It is worth noting that conventional AICD/ACID calculations discard the asymmetric component of the current-density anisotropy tensor. As reported in previous literature, this built-in approximation may yield unreliable or even erroneous trends for certain delocalised π-systems.^52,53^ For this reason, we do not draw quantitative conclusions solely relying on AICD visualisations.

Complementing the AICD results, NICS maps calculated 1 Å above the corrole ring plane and from the side view are shown in Fig. 3. At the macrocyclic center, the NICS values change slightly from −86 ppm (Au-C) to −85 ppm (Au-C-F), and from −67 ppm (Ag-C) to −66 ppm (Ag-C-F). Au-based corroles possess larger absolute NICS values than Ag analogues, consistent with the AICD observation of stronger aromatic ring-current in Au-corrole systems. The NICS values at peripheral positions remain relatively stable across all compounds, ranging from −15 ppm to −16 ppm.

Collectively, both AICD ring-current visualization and NICS aromaticity descriptors support that Au(iii) corroles exhibit stronger macrocyclic aromaticity than Ag(iii) analogues, and fluorine substitution disperses delocalized π-electrons and weakens the overall aromatic character for both metal series.

### 3.8 Analysis of weak interactions

It should be noted that the investigated *meso*-unsubstituted corrole frameworks are structurally rigid; intramolecular weak interactions characterised by IRI cannot induce observable conformational changes for these isolated molecules, and the F–F contacts occurring in fluorinated derivatives are comparatively mild. This IRI analysis is not intended to interpret conformational behaviour. Instead, it serves to map subtle redistribution of intramolecular non-covalent electronic features triggered by β-fluorine substitution. Even though these weak interactions barely alter gas-phase geometry, the modified electron-density distribution may indirectly influence condensed-phase intermolecular packing and excited-state relaxation properties.

In this work, the interaction region indicator method was used to visualize the weak interactions for four compounds, as shown in Fig. 5.

**Fig. 5:**
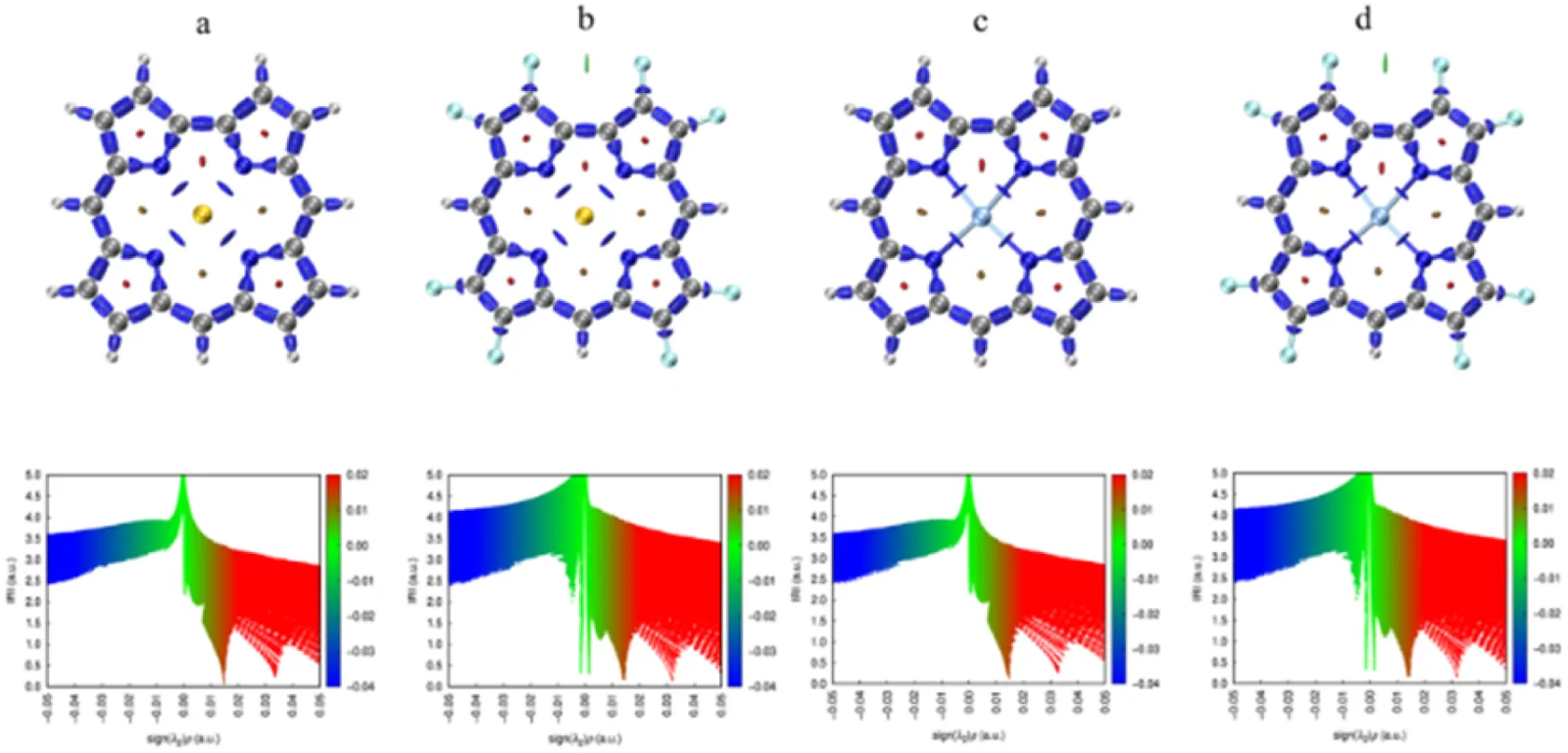
Weak interaction diagrams and scatter plots of four compounds, (a) Au-C; (b) Au-C-F; (c) Ag-C; (d) Ag-C-F.

One technical caveat should be noted: when effective core potentials are employed for heavy-metal atoms, core-electron density reconstruction *via* the Gaussian WFX subroutine can potentially produce artefacts in density-derived descriptors such as IRI in regions very close to metal atomic cores. Accordingly, our analysis below focuses exclusively on weak-interaction features distributed over the corrole macrocycle and β-fluorine sites, and we avoid interpreting signal located immediately adjacent to the Au and Ag nuclei.

Fig. 5 depicts the weak-interaction diagrams and scatter plots for four compounds: Au-corrole, Au-corrole-F, Ag-corrole, and Ag-corrole-F. These diagrams are generated *via* the interaction region indicator method.^54^ The isosurfaces of distinct colors in the figures represent different interaction types, while the scatter plots offer information regarding the compound interactions from a complementary perspective.

In Fig. 5, the green regions signify the presence of van der Waals forces or extremely weak hydrogen bonds. Such interactions are prominent in these regions of the molecule. The red regions denote the existence of certain steric hindrance effects. Regions with a deeper red color imply stronger steric-hindrance, which can impact the molecular spatial conformation and chemical reactivity. The blue regions represent significant attractive forces, and these forces are crucial for the molecule's stability and intermolecular interactions. The fully blue regions indicate the presence of relatively strong non-covalent interactions, where the electron density reaches ≥0.04 a.u., or chemical-bond interactions with an electron density significantly greater than 0.04 a.u. These regions are the key sites for bonding or strong interactions within the molecule. By analyzing the distribution of these colored regions, one can intuitively understand the interaction characteristics of different regions within the molecule, providing an essential basis for studying its chemical bonds and weak interactions. When fluorine atoms are introduced into the system, the color distribution in the figure changes remarkably. Near the fluorine atoms, the alteration of the green regions indicates that the van der Waals forces are affected, resulting in changes to the strength or range of these weak interactions.

In the scatter plot, the distribution of scatter points reflects certain quantitative characteristics of the relevant interactions. A relatively concentrated scatter-point distribution indicates that, for a specific interaction type, the variation range of the corresponding parameters is narrow, suggesting a relatively homogeneous interaction in the relevant molecular regions. Conversely, a more dispersed scatter-point distribution implies a wide variation range of interaction parameters, indicating a more complex and diverse interaction pattern in that molecular region.

## 4 Conclusions

In summary, comprehensive DFT calculations at the M06-2X-GD3/def2-TZVP-SMD(acetonitrile) level have been performed for four *meso*-unsubstituted metallocorrole complexes, namely Au-C, Au-C-F, Ag-C and Ag-C-F, to disentangle the individual and synergistic modulation effects originating from the central trivalent metal ion (Au^3+^*versus* Ag^3+^) and peripheral β-fluorination. Multiple complementary theoretical descriptors, including bond-order metrics, atomic population analysis, conceptual DFT reactivity indices, frontier-molecular-orbital decomposition, LOL-π, multicenter aromatic bond order, AICD ring-current visualization together with NICS aromaticity evaluation, and IRI weak-interaction analysis, have been jointly employed to characterise bonding features, charge redistribution, π-electron delocalization, intrinsic chemical reactivity and intramolecular non-covalent interactions.

Bond-order comparisons demonstrate that N–Au coordination bonds exhibit stronger orbital overlap and higher bonding robustness than N–Ag bonds under identical computational conditions. Upon β-fluorination, slight reduction in metal–nitrogen orbital coupling occurs for both Au- and Ag-corrole systems, driven by the strong electron-withdrawing character of fluorine atoms. Atomic-charge analysis reveals that fluorine substitution elevates positive charge density on the central metal ions, while pyrrolic nitrogen atoms remain negatively charged with moderate charge rearrangement over the macrocyclic framework. Wavefunction stability tests for *meso*-unsubstituted Ag-C and Ag-C-F confirm stable restricted closed-shell SCF solutions without spontaneous spin-symmetry breaking. This observation is specific to our *meso*-unsubstituted models and does not contradict reported ligand-non-innocent behaviour in *meso*-triaryl β-fluorinated Ag-corrole derivatives in literature, highlighting the decisive influence of *meso*-substitution on the electronic ground-state of silver corroles.

Conceptual DFT and frontier-orbital analyses show that fluorination increases the HOMO–LUMO energy gap, raises ionization potential and electrophilicity index, and suppresses nucleophilic character for both Au- and Ag-corroles. Such trends indicate enhanced electron-accepting capability together with increased chemical hardness and improved kinetic resistance to chemical perturbation after fluorine substitution. Under equivalent substitution environments, Au-corroles possess larger HOMO–LUMO gaps compared with their Ag analogues, which originates from the intrinsic difference between Au 5d and Ag 4d atomic orbitals as well as their distinct Pauling electronegativity. Combined AICD ring-current, multicenter bond-order and NICS results consistently prove that Au(iii)-corroles display stronger macrocyclic aromaticity relative to Ag(iii) analogues; β-fluorination disperses delocalized π-electrons and weakens the overall aromaticity for both metal series. IRI visualisation further illustrates that fluorine substituents perturb the distribution and intensity of intramolecular van der Waals interactions within the corrole skeleton.

It should be emphasised that all calculations in this work are based on *meso*-unsubstituted corrole model systems. The electronic-structure trends obtained here cannot be directly extrapolated to the widely-studied *meso*-triaryl corroles without careful consideration, since *meso*-aryl substituents are known to profoundly alter orbital energies and electronic configurations. Nevertheless, the present work provides a systematic quantitative baseline dataset that isolates the intrinsic effects of metal identity and β-fluorination, free from *meso*-aryl perturbation. These computational insights can serve as useful references for understanding the photophysical properties, singlet-oxygen photosensitisation and ROS-related reactivity of Au/Ag corrole-based functional systems. For systems requiring high intrinsic kinetic robustness and strong electrophilic character, fluorinated Au(iii) corrole represents a preferable candidate; Ag-corrole analogues with narrower HOMO–LUMO gaps may be more suitable for electron-transfer-related functional scenarios. Further experimental investigations are required to validate these theoretical predictions for corrole-derived functional materials.

## Author contributions

Yilei Chen: methodology, investigation, writing – original draft, writing – review & editing. Lin Dong: visualization, data curation.

## Conflicts of interest

The authors declare that there are no conflicts of interest.

## Supplementary Material

RA-OLF-D6RA07081G-s001

RA-OLF-D6RA07081G-s002

RA-OLF-D6RA07081G-s003

## Data Availability

All raw data, computational files and supplementary information (SI) results supporting the findings of this work are available from the corresponding author on reasonable request. Supplementary information is available. See DOI: https://doi.org/10.1039/d6ra07081g.
