# Supplementary material for: Fluorine substitution vs. central metal regulation: electronic properties and intrinsic reactivity of Au(iii)- and Ag(iii)-coordinated corroles from comprehensive DFT calculations
Source: RSC Adv. 2026 Oct 12;16(47):62494–503. doi: 10.1039/d6ra07081g (PMC13657201; doi:10.1039/d6ra07081g)
Supplement: Supplementary file 1 [file RA-016-D6RA07081G-s002.pdf]

Supplementary Information (SI) for RSC Advances.
This journal is © The Royal Society of Chemistry

Supporting Information

Fluorine Substitution vs. Central Metal Regulation: Electronic Properties and Intrinsic Reactivity of Au(III)- and Ag(III)-Coordinated Corroles from Comprehensive DFT Calculations

Table of contents

Section S1 Optimized Cartesian coordinates (xyz) for Au-C, Au-C-F, Ag-C, Ag-C-F S2

Section S2 Calculated energetic parameters, frequency analysis results S7

Section S3 Wavefunction-stability analysis raw outputs for Ag-C and Ag-C-F S8

Section S4 Extended discussion for HOMO-2 and LUMO+2 (moved from main-text Section 3.6) S8

Section S5 General theoretical background for IRI analysis (moved from main-text Section 3.8) S9

Section S6 Supplementary frontier-orbital plots at reduced isovalue threshold S9

Figure S2 Frontier-orbital diagrams plotted at low isovalue cutoff S10

Section S1 Optimized Cartesian coordinates (xyz) for Au-C, Au-C-F, Ag-C, Ag-C-F

Au-C:

C	0.00000000	3.48192300	2.28005800
C	0.00000000	2.56204700	3.28447600
C	0.00000000	1.25009000	2.68403500
N	0.00000000	1.43977000	1.33854300
C	0.00000000	2.78044800	1.02030300
C	0.00000000	1.82635300	-3.61885800

C	0.00000000	2.98089000	-2.84894900
C	0.00000000	2.60142400	-1.47255500
N	0.00000000	1.24689700	-1.47646700
C	0.00000000	0.71848900	-2.73154200
C	0.00000000	3.32595800	-0.26109200
C	0.00000000	-2.98089000	-2.84894900
C	0.00000000	-1.82635300	-3.61885800
C	0.00000000	-0.71848900	-2.73154200
N	0.00000000	-1.24689700	-1.47646700
C	0.00000000	-2.60142400	-1.47255500
C	0.00000000	-2.56204700	3.28447600
C	0.00000000	-3.48192300	2.28005800
C	0.00000000	-2.78044800	1.02030300
N	0.00000000	-1.43977000	1.33854300
C	0.00000000	-1.25009000	2.68403500
C	0.00000000	0.00000000	3.31455200
C	0.00000000	-3.32595800	-0.26109200
H	0.00000000	4.55750800	2.37399000

H	0.00000000	2.74630200	4.34786200
H	0.00000000	1.77117600	-4.69617100
H	0.00000000	3.99694700	-3.21284000
H	0.00000000	4.40682700	-0.32502600
H	0.00000000	-3.99694700	-3.21284000
H	0.00000000	-1.77117600	-4.69617100
H	0.00000000	-2.74630200	4.34786200
H	0.00000000	-4.55750800	2.37399000
H	0.00000000	0.00000000	4.39674100
H	0.00000000	-4.40682700	-0.32502600
Au	0.00000000	0.00000000	0.00811100

Au-C-F:

C	0.00000000	3.46508000	2.34013100
C	0.00000000	2.54906100	3.33815900
C	0.00000000	1.23853400	2.74996000
N	0.00000000	1.44013700	1.40590700
C	0.00000000	2.77723000	1.07787700
C	0.00000000	1.82653900	-3.53425000

C	0.00000000	2.97074200	-2.76740400
C	0.00000000	2.60487100	-1.39176900
N	0.00000000	1.25063500	-1.40996200
C	0.00000000	0.71257800	-2.66189200
C	0.00000000	3.33729200	-0.19240200
C	0.00000000	-2.97074200	-2.76740400
C	0.00000000	-1.82653900	-3.53425000
C	0.00000000	-0.71257800	-2.66189200
N	0.00000000	-1.25063500	-1.40996200
C	0.00000000	-2.60487100	-1.39176900
C	0.00000000	-2.54906100	3.33815900
C	0.00000000	-3.46508000	2.34013100
C	0.00000000	-2.77723000	1.07787700
N	0.00000000	-1.44013700	1.40590700
C	0.00000000	-1.23853400	2.74996000
C	0.00000000	0.00000000	3.39128800
C	0.00000000	-3.33729200	-0.19240200
H	0.00000000	4.41786300	-0.25573400

H	0.00000000	0.00000000	4.47326700
H	0.00000000	-4.41786300	-0.25573400
Au	0.00000000	0.00000000	0.07513200
F	0.00000000	-1.77858000	-4.86080200
F	0.00000000	-4.21394900	-3.23363000
F	0.00000000	1.77858000	-4.86080200
F	0.00000000	4.21394900	-3.23363000
F	0.00000000	4.78250300	2.46868900
F	0.00000000	2.78506300	4.64301500
F	0.00000000	-2.78506300	4.64301500
F	0.00000000	-4.78250300	2.46868900

Ag-C:

C	0.00000000	3.48513600	2.28151900
C	0.00000000	2.56113600	3.28246700
C	0.00000000	1.25056900	2.67637200
N	0.00000000	1.44673700	1.33348700
C	0.00000000	2.78665600	1.01915800
C	0.00000000	1.82272500	-3.61402800

C	0.00000000	2.97891800	-2.84467200
C	0.00000000	2.60161200	-1.46828000
N	0.00000000	1.24853100	-1.47049700
C	0.00000000	0.71795800	-2.72499000
C	0.00000000	3.33378900	-0.26017200
C	0.00000000	-2.97891800	-2.84467200
C	0.00000000	-1.82272500	-3.61402800
C	0.00000000	-0.71795800	-2.72499000
N	0.00000000	-1.24853100	-1.47049700
C	0.00000000	-2.60161200	-1.46828000
C	0.00000000	-2.56113600	3.28246700
C	0.00000000	-3.48513600	2.28151900
C	0.00000000	-2.78665600	1.01915800
N	0.00000000	-1.44673700	1.33348700
C	0.00000000	-1.25056900	2.67637200
C	0.00000000	0.00000000	3.30761500
C	0.00000000	-3.33378900	-0.26017200
H	0.00000000	4.56050300	2.37928600

H	0.00000000	2.74118000	4.34675300
H	0.00000000	1.76602700	-4.69138000
H	0.00000000	3.99488300	-3.20938800
H	0.00000000	4.41429500	-0.32727100
H	0.00000000	-3.99488300	-3.20938800
H	0.00000000	-1.76602700	-4.69138000
H	0.00000000	-2.74118000	4.34675300
H	0.00000000	-4.56050300	2.37928600
H	0.00000000	0.00000000	4.38970000
H	0.00000000	-4.41429500	-0.32727100
Ag	0.00000000	0.00000000	0.01102700

Ag-C-F:

C	0.00000000	3.46775200	2.34889400
C	0.00000000	2.54796400	3.34374300
C	0.00000000	1.23858600	2.75011000
N	0.00000000	1.44643400	1.40909500
C	0.00000000	2.78320400	1.08429400
C	0.00000000	1.82302900	-3.52205200

C	0.00000000	2.96902100	-2.75589500
C	0.00000000	2.60570000	-1.38068300
N	0.00000000	1.25255000	-1.39660500
C	0.00000000	0.71204600	-2.64813300
C	0.00000000	3.34486900	-0.18373300
C	0.00000000	-2.96902100	-2.75589500
C	0.00000000	-1.82302900	-3.52205200
C	0.00000000	-0.71204600	-2.64813300
N	0.00000000	-1.25255000	-1.39660500
C	0.00000000	-2.60570000	-1.38068300
C	0.00000000	-2.54796400	3.34374300
C	0.00000000	-3.46775200	2.34889400
C	0.00000000	-2.78320400	1.08429400
N	0.00000000	-1.44643400	1.40909500
C	0.00000000	-1.23858600	2.75011000
C	0.00000000	0.00000000	3.39286400
C	0.00000000	-3.34486900	-0.18373300
H	0.00000000	4.42515300	-0.24976300

H	0.00000000	0.00000000	4.47465500
H	0.00000000	-4.42515300	-0.24976300
Ag	0.00000000	0.00000000	0.08588100
F	0.00000000	-1.77550500	-4.84887300
F	0.00000000	-4.21078200	-3.22584200
F	0.00000000	1.77550500	-4.84887300
F	0.00000000	4.21078200	-3.22584200
F	0.00000000	4.78489100	2.48196800
F	0.00000000	2.77907800	4.64929600
F	0.00000000	-2.77907800	4.64929600
F	0.00000000	-4.78489100	2.48196800

Section S2 Calculated energetic parameters, frequency analysis

Table S1. Summary of calculated total electronic energies, enthalpies, Gibbs free energies and frequency-check outcomes at M06-2X/def2-TZVP level.

**Complex**	**Total electronic energy (Hartree)**	**Enthalpy H (Hartree)**	**Gibbs free energy G (Hartree)**	**Imaginary frequency**
Au-C	-1084.9983	-1084.9973	-1085.0566	None

Complex	Total electronic energy (Hartree)	Enthalpy H (Hartree)	Gibbs free energy G (Hartree)	Imaginary frequency
Au-C-F	-1878.9930	-1878.9920	-1879.0693	None
Ag-C	-1096.2305	-1096.2295	-1096.2892	None
Ag-C-F	-1890.2263	-1890.2254	-1890.3033	None

Section S3 Wavefunction-stability analysis raw outputs for Ag-C and Ag-C-F

Computational note: Wavefunction-stability calculations were performed at the M06-2X/def2-TZVP level using Gaussian keyword 'stable', starting from the respective optimized closed-shell restricted geometries.

Ag-C:

```
The wavefunction is stable under the perturbations considered.
```

Ag-C-F:

```
The wavefunction is stable under the perturbations considered.
```

Note: Only key excerpts are provided in this Supporting Information; full log files are available upon reasonable request.

Section S4 Extended discussion for HOMO-2 and LUMO+2 (moved from main-text Section 3.6)

Although the Gouterman four-orbital set (HOMO-1, HOMO, LUMO, LUMO+1) dominates the low-energy optical excitation behaviour of corrole macrocycles, higher-lying and lower-lying frontier orbitals such as HOMO-2 and LUMO+2 may still participate in certain redox-based electron-transfer processes and metal-centred excited-state formation. For completeness, the atomic-orbital composition contributions for HOMO-2 and LUMO+2 of the four studied complexes are retained in Table 5 within the main manuscript.

Nevertheless, these orbitals are not central to the core chemical conclusions of the present work. Therefore, detailed interpretive discussion regarding HOMO-2 and LUMO+2 has been relocated to Supporting Information, while the main-text analysis focuses on the Gouterman four-orbital manifold. Readers are reminded that orbital isosurface visual appearance strongly depends on the isovalue cutoff adopted during plotting; visual inspection alone cannot replace quantitative orbital-component decomposition data.

Section S5 General theoretical background for IRI analysis (moved from main-text Section 3.8)

The Interaction Region Indicator (IRI) is a real-space wavefunction-based descriptor used for visualising non-covalent interactions within molecular systems. It is constructed from the electron density and its gradient, and it can distinguish attractive,

repulsive and van-der-Waals-type weak-interaction regions without requiring bond definitions in advance.

Different interaction types are discriminated by means of the sign of the second eigenvalue of the electron-density Hessian matrix multiplied by electron density (sign(λ₂)ρ), which is represented by a colour scale on IRI scatter plots and isosurfaces: green regions correspond to typical van-der-Waals weak interactions; blue represents strong attractive interactions; red indicates steric repulsive contacts.

For calculations involving heavy-metal atoms treated with effective core potentials, core-electron density is reconstructed in post-processing via the Gaussian WFX module. This reconstruction procedure may introduce numerical artefacts in regions extremely close to the metal nuclear position, where density derivatives are evaluated. As stated in the main-text Section 3.8, our chemical interpretation of IRI results is restricted to intramolecular interaction zones located on the corrole π-macrocycle and peripheral β-fluorine substituents; features immediately adjacent to Au and Ag atomic cores are not interpreted in this work.

Section S6 Supplementary frontier-orbital plots at reduced isovalue threshold

The visual appearance of molecular-orbital isosurfaces is highly sensitive to the isovalue cutoff parameter used during plotting. In the main-text Figure 1, a relatively high isovalue was adopted for compact figure layout. At high isovalue, orbital c

ontributions with moderate percentage weight may become visually suppressed, leading to an apparent mismatch between orbital graphics and quantitative atomic-orbital decomposition percentages listed in Table 5.

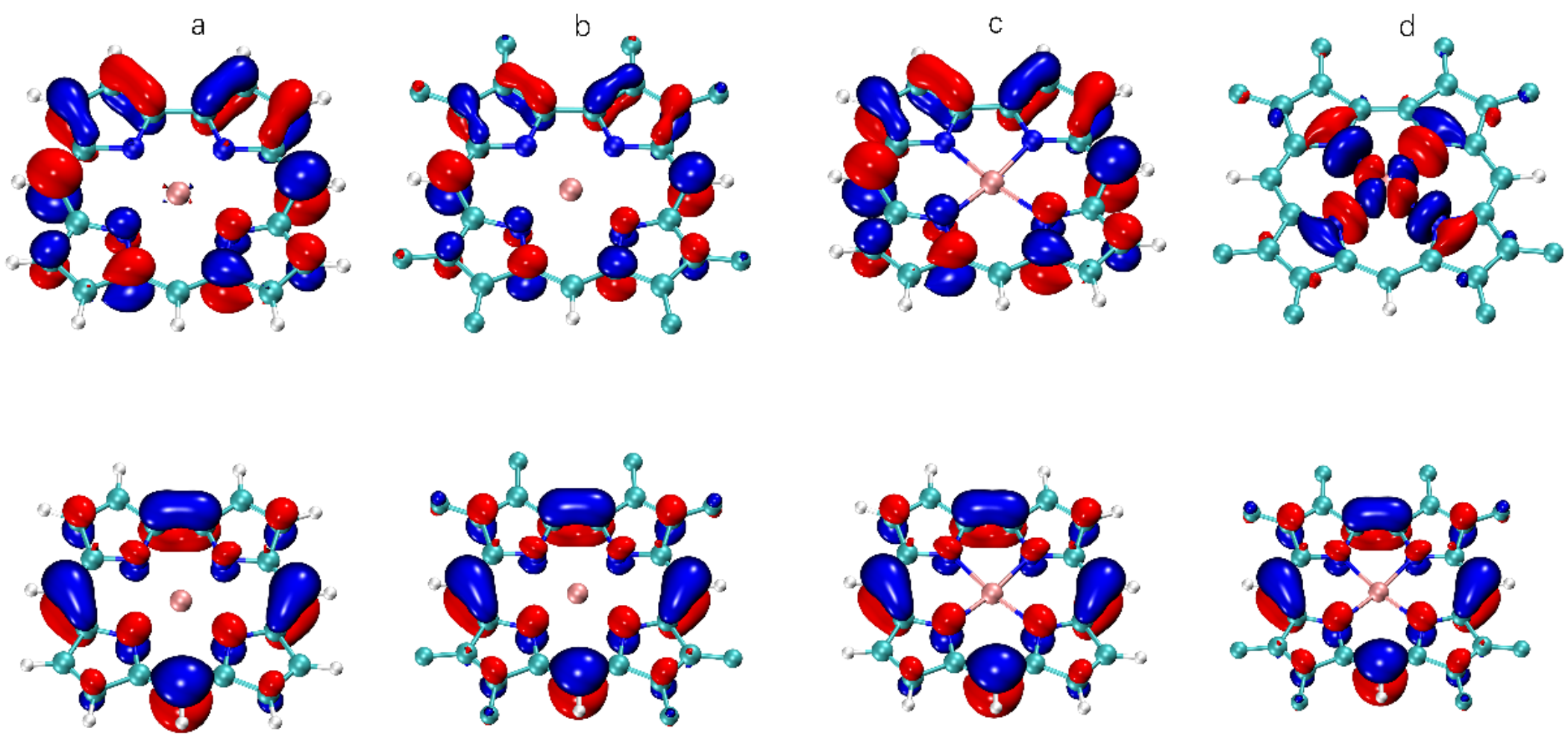

Figure S1: Frontier-orbital diagrams plotted at low isovalue cutoff. (a) Au-C; (b) Au-C-F; (c) Ag-C; (d) Ag-C-F.

Orbitals are plotted with a reduced isovalue to reveal weaker orbital lobes that are hidden under the higher isovalue condition used in main-text Figure 1.
