# Supplementary material for: Fluorine substitution vs. central metal regulation: electronic properties and intrinsic reactivity of Au(iii)- and Ag(iii)-coordinated corroles from comprehensive DFT calculations
Source: RSC Adv. 2026 Oct 12;16(47):62494–503. doi: 10.1039/d6ra07081g (PMC13657201; doi:10.1039/d6ra07081g)

Supplementary Information (SI) for RSC Advances.
This journal is © The Royal Society of Chemistry

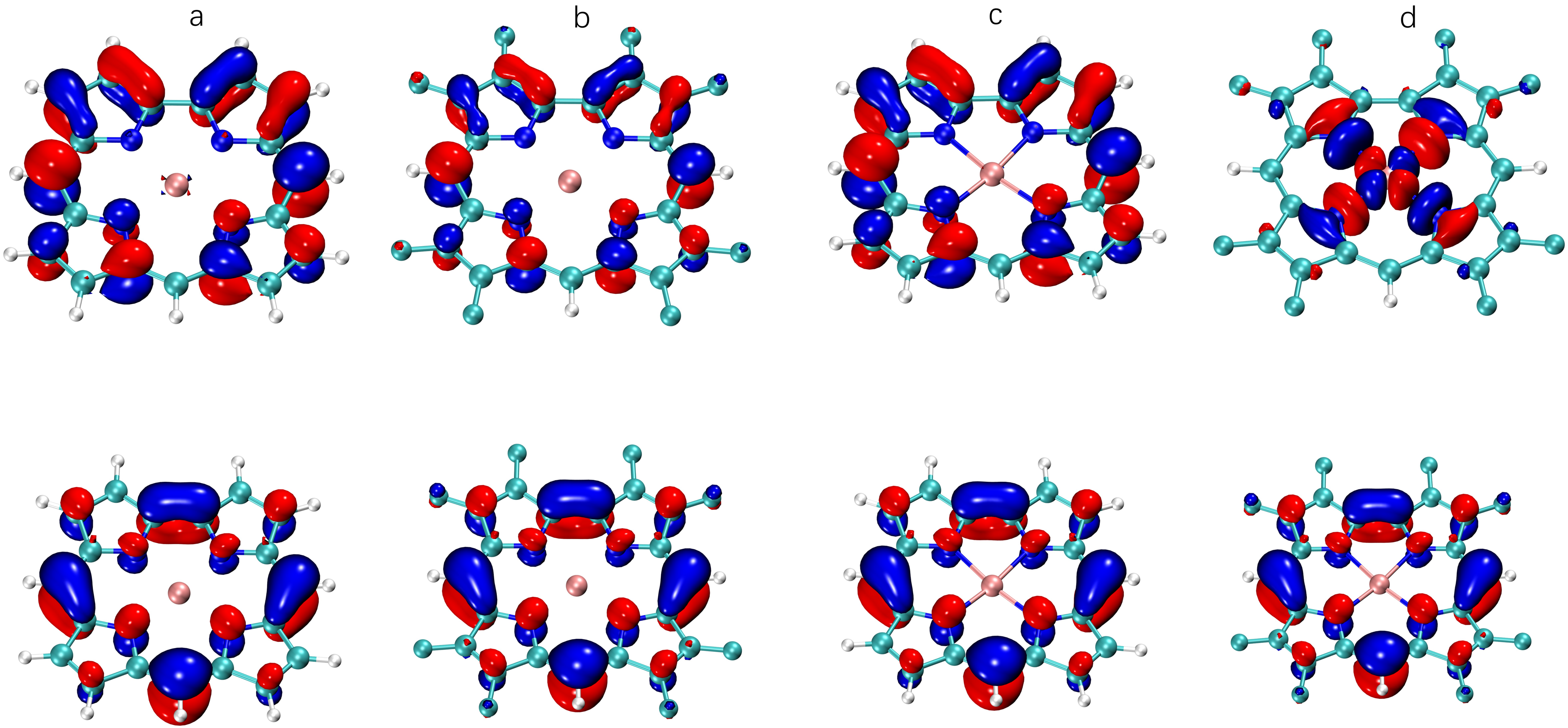

Supplement: Supplementary file 2 [file RA-016-D6RA07081G-s003.pdf]
